# Metastatic Carotid Body Paraganglioma Following Three Decades of Indolent Growth: A Case Report

**DOI:** 10.7759/cureus.115356

**Published:** 2026-08-28

**Authors:** Erik L Parkhurst, Amar Hamad

**Affiliations:** 1 International Program, Universidad Autónoma de Guadalajara (UAG) School of Medicine, Zapopan, MEX; 2 Hematology and Medical Oncology, Affiliated Oncologists, Chicago Ridge, USA; 3 Hematology and Medical Oncology, Christ Medical Center, Oak Lawn, USA; 4 Hematology and Medical Oncology, University of Illinois Chicago, Chicago, USA

**Keywords:** carotid body paraganglioma, head and neck paraganglioma, metastatic paraganglioma, multiorgan metastases, neuroendocrine tumor

## Abstract

Paragangliomas (PGLs) are rare neuroendocrine tumors arising from extra-adrenal chromaffin cells, with head and neck paragangliomas (HNPGLs) accounting for only a small proportion of all cases. Although most HNPGLs are benign, slow-growing, and nonfunctional, a minority develop metastatic disease, for which reliable histopathologic predictors remain lacking. We report the case of a 63-year-old man who presented with a progressively enlarging left-sided neck mass that had been present for more than 30 years before evaluation. Imaging demonstrated a carotid body paraganglioma with cervical lymph node involvement and distant suspected metastases to the thoracic and lumbar spine, lung, salivary gland, shoulder soft tissue, and small bowel. Histopathology confirmed paraganglioma with classic Zellballen architecture and positive staining for synaptophysin, chromogranin-A, CD56, GATA3, and S100-positive sustentacular cells. Given the patient's minimal symptoms despite extensive metastatic disease, a multidisciplinary team initially pursued active surveillance with serial multimodal imaging. Radiographic progression over time prompted initiation of systemic therapy with cabozantinib, which resulted in decreased metabolic activity of the primary tumor and no fluorodeoxyglucose-avid evidence of metastatic disease on follow-up imaging. This case highlights the unpredictable biological behavior of carotid body paragangliomas, demonstrating that longstanding, minimally symptomatic tumors may ultimately progress to widespread metastatic disease. It further emphasizes the importance of prolonged surveillance, multimodal imaging, multidisciplinary management, and individualized treatment strategies for patients with metastatic HNPGLs in the absence of reliable predictors of malignant potential.

## Introduction

Paragangliomas (PGLs) are rare neoplasms that arise from a group of neuroendocrine cells called chromaffin cells, also known as pheochromocytes, which are derived from the neural crest. Chromaffin cells are mostly found in the adrenal medulla (~90%), with additional localization in the ganglia of the sympathetic nervous system (up to ~10%) and other various parts of the body via the parasympathetic ganglia (up to ~10%) [1]. These cells secrete catecholamines such as epinephrine (adrenaline) and norepinephrine (noradrenaline) into the bloodstream. Pheochromocytomas arise from adrenal medullary chromaffin cells; paragangliomas are extra-adrenal paragangliomas. Paragangliomas can be further classified into two subtypes: sympathetic paragangliomas, which develop along the sympathetic trunk, and head and neck paragangliomas (HNPGLs), so named because most parasympathetic paragangliomas occur in the head and neck region [2,3]. Localization matters, however, because sympathetic paragangliomas typically produce catecholamines, whereas parasympathetic paragangliomas (rarer) are usually nonfunctional [4]. 

PGLs have an estimated annual incidence rate of 0.19 to 0.8 per 100,000 people, with HNPGLs accounting for approximately 3-20% of all PGLs [5,6]. Prior literature has noted PGL as the disease of 10% (10% metastatic, 10 % recurring, 10% extra-adrenal, 10% pediatric, 10% familial), however, new diagnostic techniques have illustrated that up to 36% of patients develop metastatic disease (depending on tumor type), familial history is now about 30%, and extra-adrenal tumors have been reported in 15-20% of patients [7,8]. The majority of PGL cases have an unknown underlying dysfunction and are typically referred to as sporadic, but there have been at least eight germline mutations are associated with PGL (RET: multiple endocrine neoplasia (MEN) type 2; VHL: von Hippel-Lindau disease; NF1: neurofibromatosis type 1; succinate dehydrogenase complex subunit (SDH-B, SDH-C, SDH-D): familial PGL syndrome; SDHAF1: familial PGL syndrome 2; TMEM127: familial PGL) [8-11]. Correlating patients’ clinical presentations with their genetic backgrounds has been shown to provide distinct patterns in metastatic potential, catecholamine expression, tumor location, and ultrastructural features [2]. Patients with F1, MEN, and VHL mutations rarely develop metastases, while patients with SDH-B mutations develop metastases much more frequently (up to 50-97%) [12-14]. HNPGLs are predominantly associated with the pseudohypoxia cluster classification of tumors and germline SDH-D and SDH-B (genes encoding succinate dehydrogenase subunits B and D) variants [15].

Metastasis from PGL is diagnosed by the presence of chromaffin tumors in locations where chromaffin cells are not usually present. Tissue and vascular invasion are sometimes involved, but do not necessarily correlate with disease severity or prognosis. This means that metastatic potential cannot be accurately predicted or evaluated by vascularization status and mitotic rate. Additionally, there is no single, definitive marker or predictor that can reliably determine with certainty whether a PGL will metastasize [2,15]. Clinical signs and symptoms of PGLs are variable and non-specific, making appropriate diagnosis and timely management difficult. In general, PGLs are highly curable by surgery, but the clouded prognosis of metastasis makes regular monitoring crucial to limiting significant complications and adverse outcomes [16-18]. Recent opinions have suggested a more individualized treatment approach given post-operative morbidities and advances in radiotherapy and chemotherapy, which have shown impressive results on PGLs and HNPGLs [19-22]. 

In this report, we highlight a case of carotid body paraganglioma with metastatic involvement of the spine, salivary gland, lungs, shoulder, and small bowel in a male patient with gradually increasing left-sided neck swelling for over 30 years.

## Case presentation

This case describes a 63-year-old man with no noted prior medical history who initially presented for evaluation of unilateral left-sided neck swelling that was first noticed 30 years ago but recently increased in size, which prompted referral by his dentist for primary evaluation. He had noted social and family constraints that limited his ability to seek prior evaluation but reported minor limitations with natural neck movements and rotation. No reported medication-managed comorbidities, history of smoking, history of trauma, or relevant family history. A CT neck in the winter of treatment year 1 (initial start date) post-dental examination demonstrated enlarged left-sided level II, III, IV lymph nodes measuring 2.2 x 2 cm with mild adjacent fat stranding. A mass was centered at the left carotid bifurcation, measuring 3.6 x 2.5 x 5.1 cm. Additionally, there were multiple enlarged lymph nodes at the sternocleidomastoid muscle along with a T1 vertebral lesion, measuring 1.8 x 1.6 x 1.3 cm. The patient also had symmetric enlargement of the left laryngeal ventricle, suggestive of vocal cord paralysis. Results of biochemical evaluation with plasma-free metanephrines, urinary fractionated metanephrines, methoxytyramine, or other catecholamine testing were not available at that time.

Subsequent biopsy of a left cervical lymph node with pathology one week later reported as a paraganglioma, 5.1 cm in size, involving lymphoid tissue. Flow cytometry showed no evidence of non-Hodgkin lymphoma (NHL), and Diff-Quik-stained smears showed singly scattered and small clusters of enlarged, rounded lesional cells with finely granular cytoplasm, enlarged nuclei with smooth nuclear contours, and mild hyperchromasia with fine chromatin, in a background of scattered polymorphous-appearing lymphocytes. Core biopsy sections showed a portion of lymph node with adjacent tumor composed of nests of polygonal cells with granular cytoplasm arranged in a Zellballen pattern with enlarged smooth nuclei and inconspicuous nucleoli. Remnants of lymphoid tissue, including scattered germinal centers, were also seen within the tumor. The tumor also showed increased mitotic activity. Immunoperoxidase stains on the core biopsy showed the lesional cells were positive for synaptophysin, chromogranin-A, CD56, and GATA3 (variable), and negative for keratin AE1/AE3. S100 stain highlighted scattered sustentacular cells. Ki-67 showed a proliferative index of 60-70%.

Patient established otolaryngology care in the late winter of year 1 and had a CT head that did not show any acute intracranial process. A PET scan the same month demonstrated a hypermetabolic heterogeneous soft tissue mass at the left carotid bifurcation that was consistent with the given history of carotid body tumor. The multiple hypermetabolic left cervical level II-IV lymphadenopathies were consistent with the given history of biopsy-proven paraganglioma. The hypermetabolic focus in the second part of the duodenum was nonspecific, and malignancy could not be entirely excluded. Additionally, there were multiple hypermetabolic osseous lesions involving the T1 vertebral body, left pedicle of the L4 vertebra, right iliac bone, and right sacrum that were consistent with osseous metastatic disease. Routine complete blood count (CBC) was normal, and a follow-up CT chest-abdomen-pelvis noted a 1.3 cm soft tissue nodule in the second portion of the duodenum, sclerotic foci in the T1 and L4 vertebral bodies suspicious for metastases, and diverticulosis without evidence of diverticulitis.

One month later, MRI of the thoracic and lumbar spine showed a marrow-replacing lesion within the T1 vertebral body, compatible with osseous metastatic disease with suspected early epidural extension of tumor (Epidural Spinal Cord Compression Scale (also called the Bilsky scale) (ESCC) Grade 1A) [23]. There was also a low T1 and T2 sclerotic lesion within the left L4 pedicle measuring 1.2 x 1.8 cm. Follow-up biopsy of the L4 left pedicular lesion in March 2023 demonstrated bone involved by the patient's previously diagnosed paraganglioma. The biopsy contained two small bone cores with thickened trabeculae and sheets of infiltrating cells with large cell size, abundant pink cytoplasm, and small nuclei. The cells were positive for immunostains chromogranin-A and synaptophysin. S100 showed a positive staining pattern consistent with sustentacular cells. Ki-67 was noncontributory due to decalcification of the specimen.

In the spring of year 1, the multidisciplinary team, including otolaryngology and oncology specialists, discussed possible management plans, including a conservative approach with observation and frequent imaging, with treatment initiation deferred until the patient became symptomatic. The patient agreed to the more conservative plan at that time and was regularly seen in the office without notable complaints.

Follow-up PET-CT in summer of year 1 demonstrated interval decrease in hypermetabolic activity of the soft tissue mass in the left carotid bifurcation, left cervical lymphadenopathy, hypermetabolic activity in the second part of the duodenum compared to the previous scan in the winter of year 1, along with near complete resolution of multiple hypermetabolic osseous lesions involving the T1 vertebral body, left pedicle of L5 vertebra, right iliac bone and right sacrum. Patient continued to deny any active symptoms and had no complaints at that time.

Another repeat PET-CT in the winter of year 2 showed an interval decrease in hypermetabolic activity of the soft-tissue mass at the left carotid bifurcation, along with multiple left cervical lymphadenopathy and multiple osseous lesions compared to the previous scan. There was also interval development of a new mildly hypermetabolic solid nodule in the right lateral upper lobe of the lung measuring approximately 1.0 cm of indeterminate etiology, malignancy versus reactive. Patient also had interval development of a hypermetabolic focus in the right cervical level II region, nonspecific in origin and may have represented a hypermetabolic lymph node, reactive versus metastatic lymphadenopathy. The scan also noted interval development of a hypermetabolic soft-tissue nodule in the subcutaneous fat pad of the right posterior shoulder, with a nonspecific infectious/inflammatory etiology versus malignancy/metastatic soft-tissue deposit. Finally, there was an interval increase in hypermetabolic activity of the second part of the duodenum. 

The patient was referred to a plastic surgery specialist in the late winter of year 2 due to the subcutaneous fat pad of the right posterior shoulder found on previous imaging and underwent wide excisional biopsy of the tissues with subcutaneous nodule over the left mid-back that was approximately 10 cm long and 3 cm wide, along with a complex repair. Post-procedure diagnosis of Subcutaneous nodule, probable subcutaneous tissue, and questionable metastatic tumor from the paraganglioma. Pathology results noted mature adipose tissue, consistent with lipoma. The patient continued monitoring per wishes at that time.

Repeat PET-CT in the summer of year 2 showed similar left cervical metabolically active lymphadenopathy compared to previous scans, with previously faintly metabolically active lesions within the right chest, including a right posterior shoulder skin lesion and a right pleural-based pulmonary nodule, that were resolving and attributable to possible inflammatory or reactive processes rather than malignancy. The previously described stable second-portion descending duodenal activity, although slightly increased in metabolic activity, was not considered focal and was more typical of physiologic activity.

Patient was doing well following management plan, however follow-up PET-CT in the fall of year 2 demonstrated an interval increase in hypermetabolic activity of the soft tissue mass at the left carotid bifurcation, interval development of new measurable focal hypermetabolic activity corresponding to a sclerotic lesion in the left pedicle of L4 vertebra that may be representative of progression of osseous metastatic disease, an interval decrease in hypermetabolic activity of the left cervical lymphadenopathy and interval resolution of hypermetabolic focus in the right cervical region, and a near complete resolution of hypermetabolic solid nodule in the right lateral upper lobe of lung and interval resolution of hypermetabolic soft tissue nodule in the subcutaneous fat pad of right posterior shoulder. Patient continued to opt for conservative management moving forward despite changes on imaging. 

MRI lumbar spine with and without contrast in the winter of year 3 demonstrated that the known osseous metastasis involving the left L4 vertebral body and pedicle had not significantly changed, along with no new evidence of additional metastasis in the lumbar spine. A repeat PET-CT one month later in the late winter of year 3 noted a further interval increase in hypermetabolic activity of the soft tissue mass at the left carotid bifurcation, left cervical lymphadenopathy, right cervical/right intraparotid lymph node/soft tissue nodule, along with multiple osseous lesions involving the T1 vertebra, L4 vertebra, and right iliac bone which were considered suspicious for progression of malignancy and/or metastatic disease.

Given changes in imaging and potential disease progression, the patient underwent nuclear medicine metaiodobenzylguanidine (MIBG) whole body imaging in the spring of year 3. Results noted activity within the salivary glands, with mild activity in the lungs, heart, liver, and bowel. There was also a small amount of activity in the kidneys and bladder. One week later, the multidisciplinary team with otolaryngology and oncology mutually agreed to start the patient on cabozantinib therapy at 40 mg every other day, given the unresectable and progressive metastatic nature of the paraganglioma.

**Figure 1:**
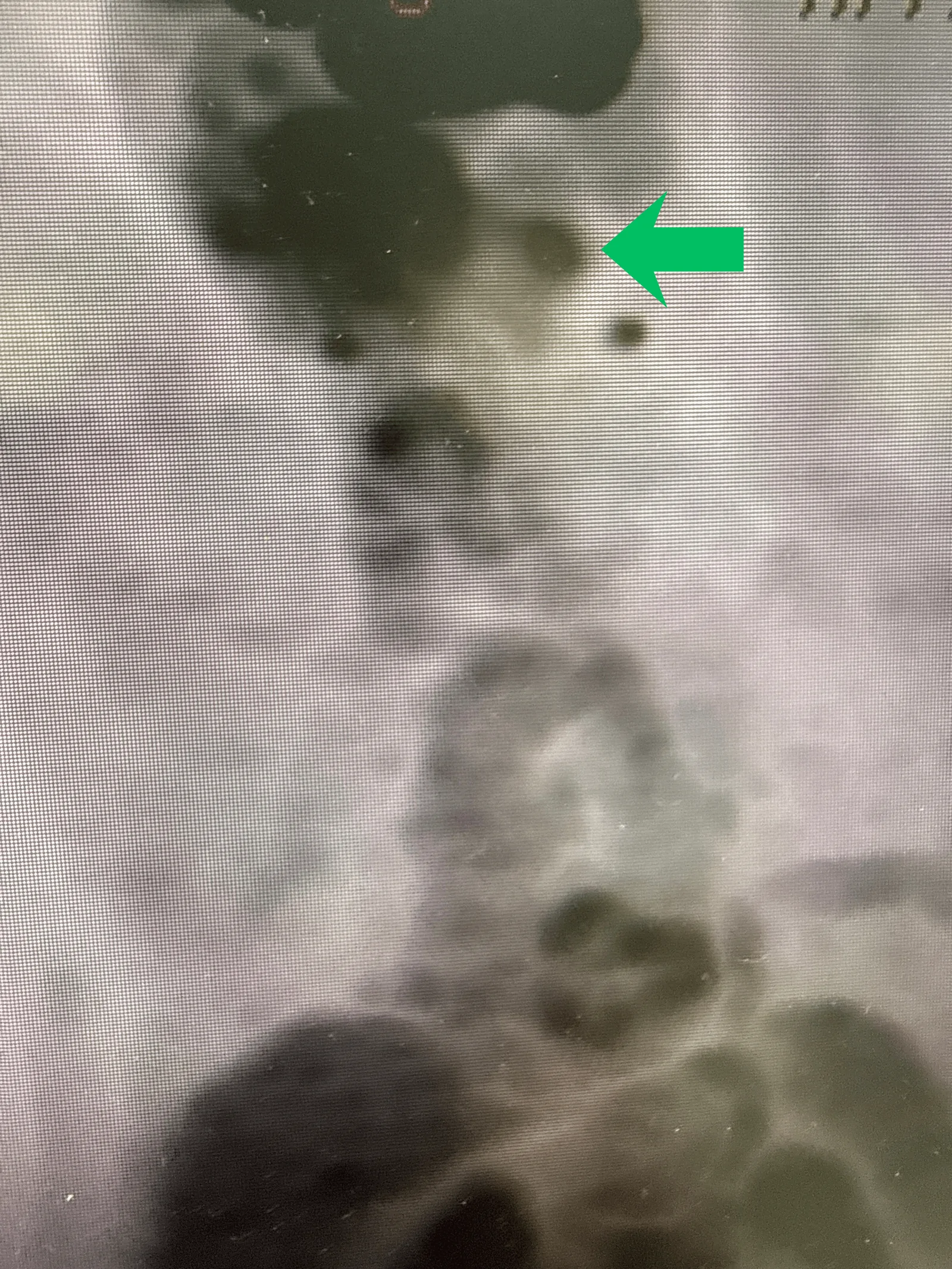
Grayscale view of a PET scan of the left carotid body PGL from late summer of year 3. PGL: paraganglioma.

**Figure 2:**
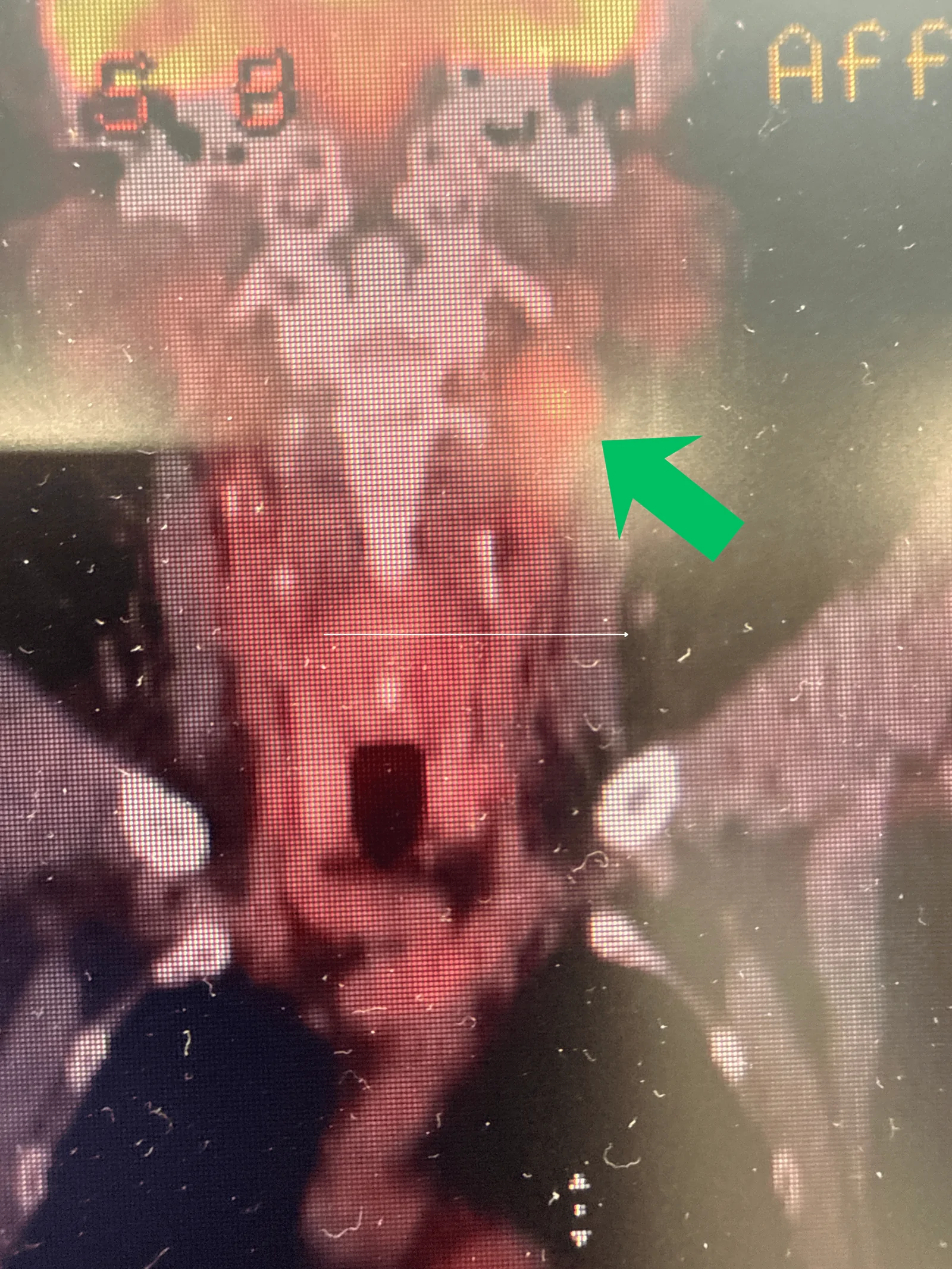
Thermal scale view of PET scan of left carotid body PGL from late summer of year 3. PGL: paraganglioma.

Patient continued cabozantinib as directed and was doing well without notable complaints until the summer of year 3, around four months after starting treatment, when he started having gastroesophageal reflux disease (GERD) symptoms, including heartburn, chest pain, trouble swallowing, a sour stomach, and regurgitation. He was sent for esophagogastroduodenoscopy (EGD) to further investigate, which showed *Helicobacter pylori* (*H. pylori*) gastritis, and the patient was started on quadruple therapy with a proton pump inhibitor (PPI), bismuth, tetracycline, and metronidazole for 14 days. Repeat PET-CT in the late summer of year 3 showed stable size but decreased metabolic activity of the left carotid body tumor, with no CT or fluorodeoxyglucose (FDG) evidence of metastasis at that time (Figures 1, 2).

Patient continued treatment with cabozantinib 40 mg and supportive fluids but was still having difficulties with GERD-like symptoms in the fall of year 3. He was restarted on quadruple therapy for two weeks with confirmatory testing at the end of treatment for *H. pylori* eradication. In mid-September, the patient was resumed on cabozantinib therapy after GERD treatment with a schedule of 40 mg every other day for two weeks and then two days on, one day off for the foreseeable future. Overall goals of the treatment plan were discussed at the time of positive response to cabozantinib, with the goal of at least eight months of continued treatment before revisiting options. Patient denied ever having issues with the most common side effects of cabozantinib, including diarrhea, fatigue, high blood pressure, mouth sores (stomatitis), significantly decreased appetite, or a hand-foot skin reaction. The side effects noted in this case reflect observational findings from a single patient's experience, not the general expected or anticipated effects (Table 1). 

**Table 1:** Chronological timeline of events. MIBG: metaiodobenzylguanidine; FDG: fluorodeoxyglucose; GERD: gastroesophageal reflux disease; EGD: esophagogastroduodenoscopy.

Timepoint	Clinical event/evaluation	Key findings/management
Winter, year 1 (start)	Initial CT neck	Left carotid bifurcation mass measuring 3.6 × 2.5 × 5.1 cm with cervical lymphadenopathy and T1 vertebral lesion.
Winter, year 1	Cervical lymph node biopsy	Paraganglioma with Zellballen architecture; positive for synaptophysin, chromogranin-A, CD56, and variable GATA3; Ki-67 60-70%.
Winter, year 1	PET-CT	Hypermetabolic left carotid bifurcation mass and cervical lymphadenopathy with osseous lesions involving T1, L4, right iliac bone, and right sacrum suspicious for metastatic disease.
Spring, year 1	L4 pedicular lesion biopsy	Bone involvement by previously diagnosed paraganglioma, confirming osseous metastatic disease.
Spring, year 1	Multidisciplinary evaluation	Conservative management with observation and serial imaging selected because the patient remained asymptomatic.
Summer, year 1	Follow-up PET-CT	Decreased metabolic activity of the primary mass and cervical lymphadenopathy with near-complete resolution of previously noted osseous lesions. Continued surveillance.
Winter, year 2	Follow-up PET-CT	Continued decrease in primary and cervical disease; new indeterminate pulmonary, cervical, and right posterior shoulder findings; increased duodenal activity.
Spring, year 2	Surgical evaluation of shoulder lesion	Wide excisional biopsy performed; pathology demonstrated mature adipose tissue consistent with lipoma rather than metastatic disease.
Summer, year 2	Follow-up PET-CT	Stable cervical disease; previously noted pulmonary and shoulder lesions resolving, favored to represent inflammatory/reactive findings.
Fall, year 2	Follow-up PET-CT	Increased metabolic activity of carotid bifurcation mass and new focal activity corresponding to a sclerotic L4 lesion, concerning for progression of osseous metastatic disease.
Winter, year 3	MRI lumbar spine	Stable known L4 osseous metastasis without new lumbar metastatic disease.
Winter, year 3	PET-CT	Further increased activity of the primary tumor with cervical and multiple osseous lesions suspicious for progression.
Spring, year 3	MIBG whole-body imaging	MIBG activity noted in multiple organs; imaging obtained as part of evaluation for progressive disease.
Spring, year 3	Multidisciplinary treatment decision	Given unresectable and progressive metastatic disease, cabozantinib initiated at 40 mg every other day.
Summer, year 3	Clinical follow-up	Developed GERD symptoms; EGD demonstrated *H. pylori* gastritis. Quadruple therapy initiated.
Summer, year 3	PET-CT	Stable size but decreased metabolic activity of the left carotid body tumor with no CT or FDG evidence of metastasis at that time.
Fall, Year 3	Treatment follow-up	Cabozantinib resumed following treatment for *H. pylori*, initially 40 mg every other day and subsequently adjusted to 2 days on/1 day off.

## Discussion

Head and neck PGLs (HNPGLs) are considerably rare, with estimates of incidence as low as 3% of all PGLs and accounting for only 0.5% of tumors in the region [6]. Although most reported HNPGLs are deemed benign, these tumors are considered very slow-growing and often go undetected for years, with a low metastasis rate of 6% to 19% [6,20]. A National Cancer Database report on malignant paragangliomas of the head and neck reported an estimated five-year survival rate of 76.8% in patients with metastasis to regional lymph nodes and 11.8% in patients with distant metastasis [24,25]. In the case presented here, the patient had a notable left-sided neck mass for decades that continued to grow and was formally diagnosed as a paraganglioma with metastasis, which is attributable to the likely duration of the tumor presence and lack of established care due to personal factors. 

A majority of HNPGLs are non-secretory tumors, and clinical characteristics may depend on the location of the tumor. While all PGLs have the capacity to generate catecholamines, only roughly 3% to 4% are thought to secrete them [26]. The most typical presentation involves a painless neck mass with possible symptoms of hoarseness, tinnitus, cranial nerve palsy-like neurological deficits, and ear pain. Carotid body PGLs comprise about 60% of all HNPGLs, and most associated symptoms are due to the compression exerted on the nearby cranial nerves, like the vagus and glossopharyngeal nerves [19,27-29]. The second most common location for HNPGLs is the middle ear, at about 30% of cases, but these tumors are generally more symptomatic with pulsatile tinnitus, hearing loss, and aural fullness. Middle ear HNPGLs also have a much lower metastasis risk of about 2% [30]. In the case presented here, the patient had a primary carotid body PGL but lacked any of the characteristic symptoms associated with HNPGLs, which is not necessarily atypical but rather unusual. 

Imaging is a key component in the current management protocol of HNPGLs and offers vital details towards establishing an appropriate care plan. Key information that can be extrapolated from imaging modalities includes: primary tumor localization, the presence of multiple coexisting tumors allowing differentiation between a singular mass and possible syndromic paraganglioma, and the existence of distant metastases [19,28,31]. A CT scan of PGLs appears as a solid homogeneous mass with high uptake due to hypervascularization and can offer information related to anatomical relationships with the internal carotid artery and venous return, a better understanding of the vascularization of the tumor, and guidance for surgical or chemoembolization decisions if applicable [32]. Additionally, CT imaging has a sensitivity of 80-90% and a specificity of 90% for local bone invasion, but is generally unreliable in the predicament of malignancy [31]. MRI has become a more standard imaging method in recent years to assess PGLs, assuming patients and facilities have access to reliable and affordable imaging. PGLs on MRI can be described as a solid mass with heterogeneous enhancement due to a rich vascular network with a “salt-and-pepper” appearance, which translates into rapid flow vessels (pepper) integrated in a tumoral matrix or bleeding zones with a high-intensity signal (salt) [19,33]. MRIs can provide information regarding the invasion of soft tissue and metastatic lesions with the same specificity and sensitivity as CT, but without radiation as a benefit [31]. More functional imaging methods have come to the forefront of PGL management in recent years, notably 123I-MIBG scintigraphy and PET-CT (18F-FDA or 18F-FDG). 123I-MIBG scintigraphy has limited use in metastatic PGLs, with an identification rate of 59% to 79%, but has a sensitivity of 92% to 98% for non-metastatic tumors [34]. PET-CT modalities, particularly 18F-FDG, have great utility in localizing primary paragangliomas and metastases, but sensitivity ranges from 58% to 88%, and results should be interpreted alongside all other imaging and clinical factors [34,35]. 18F-FDA PET-CT has been proven to show superiority to 123I-MIBG scintigraphy in identifying metastases and primary PGLs derived from the sympathetic nervous system, with a sensitivity between 70% and 95%, but has performed poorly for HNPGLs (from the parasympathetic nervous system) with a sensitivity of only 46% [31,35]. Next-generation imaging tools, like 68Ga-DOTATATE PET-CT, have emerged as an incredibly valuable option for evaluation of PGLs and metastases due to higher uptake related to the somatostatin receptor (SSTR) overexpression in these types of tumors, especially SSTR2, which can detect 36% more lesions than CT or MRI [36]. Its excellent results in evaluating PGLs have even led to the European Association of Nuclear Medicine's guidelines changing [37]. Still, it remains an imaging modality that is somewhat limited due to cost and availability in the United States, particularly in areas outside of major hospital networks or academic centers [38]. In the case presented here, the patient had a variety of imaging modalities due to the unique case and presence of multiple areas of metastasis. All available modalities were considered and discussed during the multidisciplinary oncology team meetings alongside the patient. Predominant imaging was PET-CT for monitoring of the paraganglioma and metastasis, with CT used as a primary diagnostic tool, MRI for bony metastasis, and MIBG for disease progression evaluation. 

No reliable markers currently exist for metastatic disease in PGL, and malignancy is diagnosed based on the presence of metastases. Various pathological markers used in other tumors have been evaluated for PGLs, including tissue invasion, high vascularization, and staining for specific proteins, such as Ki-67, but none have proven predictive or definitive [2,39,40]. Paragangliomas are composed primarily of epithelioid cells, with sparse spindle cells, and have round, hyperchromatic, and chromatin-clustering nuclei that stain positively for synaptophysin [30]. The latest World Health Organization consensus considers previously used biomarkers such as CD56, CD57, and neuron-specific enolase obsolete and identifies chromogranin-A as the most specific biomarker for paragangliomas and all other neuroendocrine neoplasms [28,41]. GATA3 has also been deemed practical for HNPGLs but shows wide variation in expression detection [42]. In the case presented here, biopsy results demonstrated positive staining for synaptophysin, chromogranin-A, CD56, and GATA3. The markedly elevated Ki-67 proliferative index of 60-70% observed in the cervical lymph node biopsy is notable and suggests substantial proliferative activity. However, Ki-67 should not be interpreted in isolation as a definitive predictor of metastatic behavior in paraganglioma. In this patient, the subsequent development and radiographic progression of metastatic disease provide independent evidence of malignant potential. The prolonged period of minimal symptoms and initial radiographic stability therefore reflects the patient's clinical course rather than necessarily indicating low tumor proliferative activity. The Ki-67 finding should consequently be interpreted alongside the patient's metastatic pattern, serial imaging, pathology, and clinical trajectory.

The mainstay of management and treatment in the recent past was centered on surgical resection of the primary tumor, but recent opinions have pushed for more individualized plans of action. These shifts reflect trends in postoperative morbidity, along with advances in chemotherapy and radiotherapy, which have substantially improved HNPGL outcomes [20-22]. Recurrence rates vary with therapeutic approach and PGL localization; however, carotid body PGLs have been suggested to have the highest recurrence rate when treated with radiotherapy, whereas surgery yields the lowest recurrence rate (excluding metastatic components) [43]. Carotid body PGLs are indolent tumors, with an average growth rate of only 10.4% per year, which has encouraged monitoring in patients with small, asymptomatic benign tumors over 60 years old; however, waiting may increase the risk of skull base and carotid artery invasion [44,45]. The Shamblin classification helps predict the difficulty of surgical intervention [46]. Determinations of stroke risk and cranial nerve injuries in relation to the Shamblin classification have also been established [47]. The Shamblin classification has been modified over the years, with a focus on the tumor's infiltrative relationship with the carotid artery and the degree of vascularity [48]. See Table 2 for additional details and for reference. Limitations of the Shamblin classification include the exclusion of PGLs that extend to the infratemporal fossa and those that involve the carotid canal or jugular foramen, but no other consensus classification systems have been developed [49,50].

**Table 2:** Modified Shamblin classifications for carotid body PGLs with additional relationships. PGL: paraganglioma. References: [46-51].

Grade/group	Tumor characteristics	Stroke risk	Cranial nerve injury correlation
Shamblin I	<4 cm in size not surrounding or infiltrating the carotid excision without difficulty	15%	7%
Shamblin II	>4 cm in size not surrounding or infiltrating the carotid excision is difficult	25%	39%
Shamblin III	>4 cm in size significantly infiltrating or surrounding the carotid vessels, notable excisional difficulty requiring vascular repair, vessel replacement, or sacrifice	60%	54%

Additional treatment measures for metastatic PGLs, in addition to surgery, include radiotherapy, chemotherapy, immunotherapy, and peptide therapy. In most cases of distant metastasis, as in the case report presented here, chemotherapy is the treatment of choice. Typical agents used in practice include cyclophosphamide, vincristine, dacarbazine, temozolomide/capecitabine, sunitinib monotherapy, and thalidomide [52]. We used cabozantinib, an antiangiogenic multi-tyrosine kinase inhibitor, in the case presented here after lengthy discussions with the patient and the multidisciplinary treatment team. This treatment, along with other tyrosine kinase inhibitors, is a new development, and recent results from a single-arm phase 2 trial of metastatic paraganglioma treatment provided promising evidence that these agents might represent a solution for treating metastatic PGLs in the near future [53]. Other new agents under evaluation target the somatostatin receptor-ligand, but results are pending [20]. Researchers investigated treatment with the mTOR inhibitor everolimus without significant results [54]. IL-13Rα2 is a significant target under consideration for new therapies because it blocks anti-tumor responses and promotes cancer progression, but its specific clinical relevance and therapeutic potential in PGLs remain areas needing more focused investigation. 

The prognosis of metastatic HNPGLs with lymph node metastasis is based on an overall survival rate after five years of approximately 76.8%, and 11.8% with distant metastasis [25]. Even with survival after treatment, quality of life can be altered permanently, such as surgical intervention leading to hoarseness due to vocal fold palsy after vagal nerve surgery, or fatigue in case of ventilatory dysfunction [55]. Anxiety and depression are also very common, similar to the overall rates of any oncologic disease [20]. No consensus exists for the duration of surveillance, but general recommendations from the British Skull Base Society dictate serial imaging for at least three years, followed by reduced follow-up thereafter, while the European Society of Endocrinology advises a 10-year minimum period of serial imaging with up to lifelong surveillance in patients with notable risk factors such as genetic predisposition [19].

## Conclusions

HNPGLs are extremely rare entities with challenging pathologies. Although the majority of these are benign, this case report highlights that a section of longstanding, minimally symptomatic tumors may still develop extensive metastatic disease. The prolonged clinical course and delayed presentation underscore the diagnostic challenges associated with non-secretory HNPGLs and the importance of maintaining clinical suspicion in patients with slowly enlarging neck masses. Serial multimodal imaging was essential for assessing disease burden and guiding management in the absence of reliable histopathological predictors of malignancy. In this patient, a conservative surveillance-based approach was initially appropriate, with subsequent transition to systemic therapy following radiographic progression. Cabozantinib was the targeted therapy of choice given its potential, albeit limited, efficacy in selected cases of unresectable metastatic PGL, as in the patient’s case. This report reinforces the need for individualized, multidisciplinary management strategies and consistent follow-up in patients with HNPGLs. Recommendations would focus on conducting further studies to harmonize treatment strategies and inform standardized clinical guidelines.
